# The impact of chlorophyllin on deoxynivalenol transport across jejunum mucosa explants obtained from adult pigs

**DOI:** 10.1007/s12550-019-00342-2

**Published:** 2019-02-02

**Authors:** Marta Mendel, Wojciech Karlik, Magdalena Chłopecka

**Affiliations:** Division of Pharmacology and Toxicology, Faculty of Veterinary Medicine, Warsaw University of Life Sciences, 8 Ciszewskiego St, Warsaw, Poland

**Keywords:** Deoxynivalenol, Chlorophyllin, Mucosa explants, Adult pigs

## Abstract

Regardless of the efforts put into preventing or reducing fungal growth, extensive mycotoxin contamination has been reported in animal feeds. In the case of pigs, one of the mycotoxins of major concern is deoxynivalenol (DON). The use of adsorbents as feed additives represents one of the strategies to control mycotoxins’ contamination in feedstuff. Therefore, the aim of the study was to verify the ability of chlorophyllin (CHL) to reduce the absorption rate of DON in swine mucosa explants. Intestine was obtained from routinely slaughtered adult pigs. The mucosa explants were studied by means of Ussing chamber technique. The effect of DON (10 and 30 μg/ml) on mucosa viability and permeability and CHL (100 μg/ml) impact on DON (30 μg/ml) absorption was verified. The results revealed that mucosa explants isolated from adult animals remained unaffected for 90 min in the presence of DON in the lower concentration (10 μg/ml). Mycotoxin in the higher dose (30 μg/ml) increased mucosa permeability (decreased transepithelial electrical resistance value) and enhanced paracellular transport of lucifer yellow and mannitol but did not affect lactate dehydrogenase leakage. The introduction of CHL neither diminished the absorption rate of DON across swine mucosa explants nor prevented the toxic effects of DON on intestine. In conclusion, the results confirm the negative effect of DON on pig jejunum mucosa. However, the toxic effect of DON was observed only when it was used in relatively high doses. A promising adsorbent agent, CHL, failed to reduce the intensity of DON transport across intestine under in vitro conditions.

## Introduction

The problem of mycotoxin occurrence in numerous food and feed is well recognized worldwide. Despite the efforts put into preventing or reducing fungal growth, extensive mycotoxin contamination in animal feed still continues to be a problem for livestock production. Aflatoxins, deoxynivalenol (DON), fumonisins, ochratoxin A, T-2 toxin, and zearalenone are the major contaminants of feed (Streit et al. 2012, 2013; Bryła et al. 2018). Among these mycotoxins DON, a type-B trichothecene produced by *Fusarium culmorum* and *F. graminearum* (European Food Safety Agency 2004), is a major concern with regard to pig health and production (Rotter 1996; Dersjant-Li et al. 2003; Pinton et al. 2009; Ghareeb et al. 2015). Data summarized by Streit et al. (2012) indicate that mycotoxins are ubiquitously present in feed material throughout Europe and that maximum contamination levels exceeding the European Union (EU) guidance values (European Commission 576/2006) are likely to occur. Noteworthy, the EU maximum levels and the United States Food and Drug Administration (FDA) advisory levels for complementary and complete feeding stuffs for pigs are 900 and 5000 μg/kg, respectively (European Commission 576/2006, FDA 2010). Contamination levels of DON in feed typically cover a wide range from less than 100 to 5000 μg/kg, but levels exceeding 10,000 μg/kg have also been reported (Grajewski et al. 2012; Rodrigues and Naehrer 2012; Bernhoft et al. 2016; Streit et al. 2013; EFSA 2017). Additionally, the co-occurrence of several mycotoxins in food and feed is a frequent observation (Streit et al. 2013; Bernhoft et al. 2016; Smith et al. 2016).

Feed contaminations caused by mycotoxins jeopardize animal health (acute animal illnesses) and productivity due to chronic low-level exposures. In swine production, DON (12–20 mg DON/kg diet) is mainly known for causing feed refusal and emesis, hemorrhage, and circulatory shock (Rotter 1996; Pinton et al. 2009). Besides, the chronic exposure to low doses of this mycotoxin (0.1–3 mg DON/kg diet) suppresses heavily the immune response and the intestinal functions, causes anorexia, reduced weight gain, and neuroendocrine changes (Rotter 1996; Cheng et al. 2006; Pinton et al. 2008).

Despite progress made in prevention through breeding of resistant varieties and improvement in agronomic practices, significant amounts of mycotoxins might be found in feed materials. There are numerous strategies to control mycotoxins’ contamination in feedstuff and to reduce livestock exposure to these toxins, e.g., milling, baking, implementation of yeast-derived products or glucomannans in animal feeding (Kabak et al. 2006; Boudergue et al. 2009). Moreover, biotransforming microorganisms which are claimed to degrade trichothecenes enzymatically or to help to maintain normal function of the gut mucosa are nowadays the most favored and the most studied approach to reduce animal exposure to mycotoxins, particularly in case of DON (Niderkorn et al. 2007; Young et al. 2007; Alassane-Kpembi et al. 2018; García et al. 2018). Another strategy consists of adsorbent materials that may bind fungal toxins in the lumen of gastrointestinal tract and, thereby, reduce their local and systemic toxicity (Boudergue et al. 2009; Awad et al. 2010; Karlovsky 2011; García et al. 2018). Unfortunately, most of the agents (e.g., natural and modified clay minerals or activated carbon) used these days fail to detoxify DON-contaminated feedstuff (Avantaggiato et al. 2004). Thus, novel substances are necessary to minimize the exposure of animals, mainly pigs, to this mycotoxin. Chlorophyllin (CHL) is a promising adsorbent which could potentially inhibit the transport of DON across the gut wall. CHL is a semi-synthetic, water-soluble sodium-copper salt of chlorophylls which is authorized for coloring foodstuffs by Community rules (category 2a) (European Union Register of Feed Additives 2018). There is some evidence of CHL ability to inhibit mycotoxin, e.g., inhibition of aflatoxin B_1_ (AFB_1_) transport across Caco-2 cell monolayer (Mata et al. 2004) and complexing of DON in phosphate buffer (Cavret et al. 2010). Consequently, the objective of this study was to verify the ability of CHL to reduce the absorption of DON in swine jejunum explants isolated from adult pigs using the Ussing chamber technique.

## Materials and methods

### Reagents

CHL, D-mannitol, D-mannitol colorimetric assay kit, disodium fumarate, l-glutamate, LDH cytotoxicity detection kit, lucifer yellow (LY), and sodium pyruvate were obtained from Sigma-Aldrich (St. Louis, USA). All inorganic salts required for the preparation of Krebs-Bicarbonate Buffer, ethanol, and glucose were purchased from Avantor (Gliwice, Poland).

Purified DON (Sigma Aldrich, St. Louis, USA) was dissolved in ethanol and stored at − 20 °C before dilution in the incubation medium. MaxSignal DON ELISA Test Kit was obtained from Bioo Scientific Corporation (Texas, USA).

### Tissue preparation

Twenty healthy adult crossbred pigs which underwent routine slaughter were used for the collection of intestinal tissues. Segments of jejunum (approx. 150 cm aboral to pylorus) were excised and immediately flushed with ice-cold (0–4 °C) Krebs-Bicarbonate Buffer (KRB) containing 108 mM NaCl, 4.7 mM KCl, 1.8 Na_2_HPO_4_, 0.4 mM KH_2_PO_4_, 15 mM NaHCO_3_, 1.2 MgSO_4_, 1.25 mM CaCl_2_, 11.5 mM glucose, 4.9 mM l-glutamate, 5.4 disodium fumarate, 4.9 mM sodium pyruvate, pH 7.4, and saturated with oxygen using a 95%/5% O_2_/CO_2_ mixture by gassing for 60 min (Ungell et al. 1992). Next, the intestine tissues were transported to the laboratory. The time between animal slaughter and mucosa explant fixation in the incubation chamber amounted to 60–80 min. Under laboratory conditions, the intestines were subjected directly to ex vivo preparation as described before (Kolf-Clauw et al. 2009; Sjöberg et al. 2013; Westerhout et al. 2014). Briefly, each intestinal tissue was cut into pieces of 10–20 cm and opened longitudinally. Then, continuously submerged under ice-cold oxygenated KRB, the serosa and both muscular layers were carefully stripped from the mucosa using a forceps. Finally, three mucosa explants were collected from each animal. The limited number of explants isolated from one animal is explained by two issues. Firstly, the intention was to work on jejunum preparations isolated from the same region of intestine, i.e., originally located within the 150 to 165 cm of the aboral to pylorus region. Secondly, in order to mount the explants in the incubation chambers latest 80 min after animal slaughter, it was possible to prepare only three explants each time. Each resulting preparation of mucosa with attached submucosa was mounted separately between two Ussing-type half chambers (1.54 cm^2^ tissue exposure area). Jejunum sheets were bathed on both luminal (mucosal) and contraluminal (serosal) surfaces in 10 ml of KRB maintained at pH 7.4 and 37 °C. Mucosa explants were continuously oxygenated on both luminal and contraluminal surfaces with 95%/5% O_2_/CO_2_ mixture delivered by a gas lift. The complete system was then preincubated for 10 min in a humidified incubator at 37 °C for equilibration of the tissue. Next, the incubation medium was replaced by fresh KRB in serosal chamber and KRB supplemented with LY at 250 μg/ml and mannitol at 18.22 μg/ml (KRB+LY+M) in mucosal chamber. During the incubation, the complete system was gently shaken on a rocker platform at 65 cycles per minute.

### Measurement of the viability and integrity of mucosa explants

The viability and permeability of swine jejunal tissue segments were assessed by measuring several markers directly after preincubation (time 0), 60 and 90 min later. The integrity of mucosa explants was assessed by measuring transepithelial electrical resistance (TEER) using Millicell ERS-2 Epithelial Volt-Ohm Meter (Merck KGaA, Darmstadt, Germany). Only intestine strips with TEER readings greater than 70 Ω cm^2^ at time 0 were used in further parts of the experiment (Westerhout et al. 2014). Moreover, the integrity and viability of the explants were assessed by measuring paracellular transport of LY and mannitol over time from the luminal to the contraluminal compartment. To verify the possible tissue damage caused by the presence of active proteases or experiment duration, the leakage of lactate dehydrogenase (LDH) to both luminal and contraluminal compartments was recorded.

### Ex vivo exposure of swine jejunum to deoxynivalenol and chlorophyllin

The preliminary study included the verification of DON solvent, i.e., ethanol (0.1%), on mucosa explants viability, integrity, and permeability. For this purpose, one intestine preparation was incubated in pure KRB and another one isolated from the same animal was incubated in KRB containing ethanol (0.1%). TEER values, LY, and mannitol flux, as well as LDH leakage were compared for both explants. Six pairs of explants obtained from six animals were used in this trial.

In the first set of experiments of the main study, the effective (toxic) concentration of DON towards mucosa explants isolated from adult pigs was determined. Three explants of jejunum mucosa were obtained from each pig and were mounted separately in Ussing-type chambers. The first strip was used as a control tissue, i.e., it underwent 10-min preincubation followed by 90 min. Incubation in KRB (serosal chamber) and KRB+LY+M with no addition of DON (mucosal chamber). Control samples were treated with ethanol (0.1%, the final concentration achieved in mucosal chamber) to exclude the impact of DON solvent on tissue viability and integrity. The second and third preparations were subjected to the 10-min preincubation in KRB which was replaced by KRB in the serosal compartment and KRB+LY+M supplemented with DON (10 or 30 μg/ml) at the mucosal site for the following 90 min. In the second set of experiments, the effect of CHL on DON penetration across mucosa explants was verified. Again, three explants of jejunum mucosa were obtained from each pig and were mounted separately in Ussing-type chambers. One jejunum preparation was used as a control tissue (no addition of DON or CHL); one was incubated in the presence of DON (30 μg/ml) and the last one in the presence of DON (30 μg/ml) and CHL (100 μg/ml) (luminal compartment) for 90 min. Finally, in the third set of experiments, the effect of CHL (100 μg/ml) on mucosa explants viability, integrity, and permeability was determined. Two explants of jejunum mucosa were obtained from each pig and were fixed separately in Ussing-type chambers. One jejunum preparation was incubated in pure medium (KRB) and the other one in the presence of CHL (100 μg/ml). The preincubation and buffers in the second and third trial were analogous to those from the first set of experiments. TEER measurement as well as sample collection (600 μl) for later LY, mannitol, LDH, and DON assays were conducted at time 0, 60, and 90 min after the onset of incubation. Experiments of the first set were conducted on mucosa explant isolated from eight animals (in total 24 explants); experiments of the second and third trial were conducted on jejunum segments obtained from six animals (in total 18 and 12 explants, respectively).

The concentration of DON was assessed by MaxSignal DON ELISA Test Kit (Bioo Scientific Corp. 2016). Sample preparation was performed according to the protocol described for serum. Prior to the determination analysis, linearity, range, and accuracy of the test were confirmed. LY was analyzed directly in samples using a FL_x_800 Microplate fluorescence reader (BioTek Instruments, Inc., Winooski, USA) at excitation wavelength 485 nm and emission wavelength 530 nm. LDH activity and mannitol concentration were determined using a Cytotoxicity Detection Kit (LDH) and D-Mannitol Colorimetric Assay Kit, respectively, (Sigma-Aldrich, St Louis, USA) according to the manufacturer’s instruction.

The results of DON, LY, and mannitol penetration across intestine mucosa explants are expressed as mass flux or the concentration of the chemical found in the serosal compartment. The activity of LDH leakage into the incubation media is expressed as a percentage of total LDH, which was analyzed after explants homogenization in ice-cold KRB with a Potter S Homogenizer (B. Braun Biotech International, Berlin, Germany) for 2 min at 1000 rpm.

### Statistical analysis

Data obtained in the whole experiment, i.e., in all three sets, were combined and analyzed together. Finally, the control group had a total of 20 replicates, tested groups: DON 10 μg/ml had 8 replicates, DON 30 μg/ml—14, DON 30 μg/ml + CHL—6, and CHL—6 replicates. Experimental results are expressed as means ± SEM. The differences between means were statistically determined using ordinary one-way ANOVA followed by Tukey’s multiple comparisons test. The results are considered statistically significant when *P* < 0.05. The analyses were performed using GraphPad Prism version 7.04 for Windows, GraphPad Software, La Jolla CA USA, “www.graphpad.com.”

## Results

### The viability and integrity of swine jejunal mucosa

**Fig. 1:**
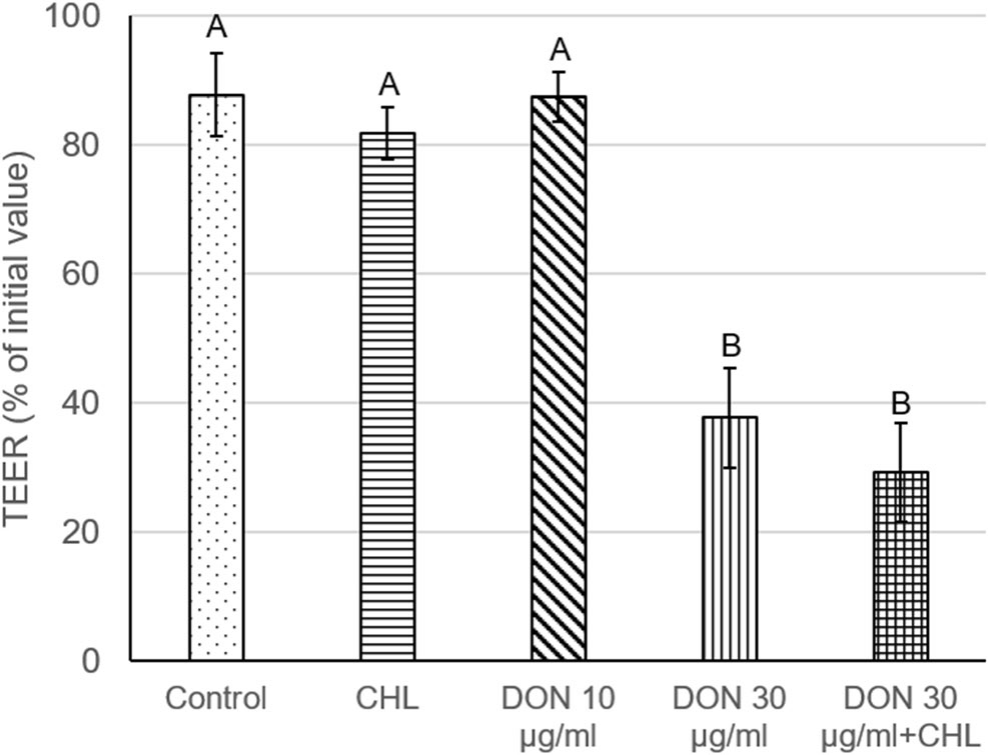
Effect of DON (0, 30, and 90 μg/ml) and DON (90 μg/ml) + CHL (100 μg/ml) on trans-epithelial electrical resistance (TEER) in swine mucosa explants after 90 min of incubation in KRB. TEER values are expressed as % of the initial TEER value measured after equilibration (preincubation). Data is expressed as mean ± SEM of *n* independent experiments, where *n* = 20 for control, *n* = 6 for CHL, *n* = 8 for DON 30 μg/ml, *n* = 14 for DON 90 μg/ml, and *n* = 6 for DON 90 μg/ml + CHL The results are considered statistically significant (different letters) when *P* < 0.05.

**Fig. 2:**
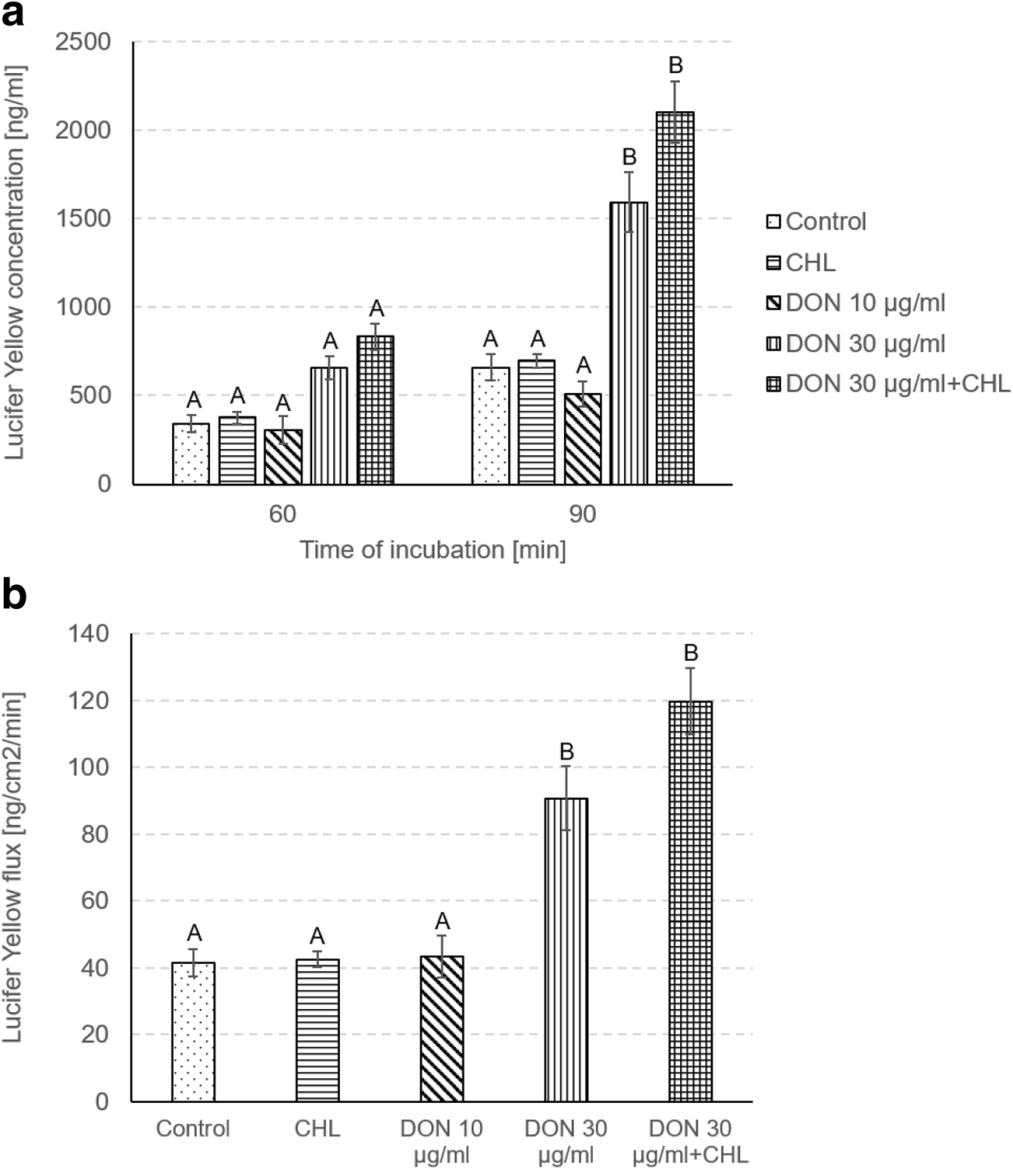
Effect of DON (0, 30, and 90 μg/ml) and DON (90 μg/ml) + CHL (100 μg/ml) on paracellular passage of lucifer yellow (LY) across intestinal explants. The rate of LY penetration is expressed as the concentration measured in the serosal compartment (**a**) or flux (**b**). Samples for LY detection were collected from the serosal reservoir after equilibration (preincubation), 60 and 90 min thereafter. LY flux was calculated for 90 min. Data is expressed as mean ± SEM of *n* independent experiments, where *n* = 20 for control, *n* = 6 for CHL, *n* = 8 for DON 30 μg/ml, *n* = 14 for DON 90 μg/ml, and *n* = 6 for DON 90 μg/ml + CHL The results are considered statistically significant (different letters) when *P* < 0.05

**Fig. 3:**
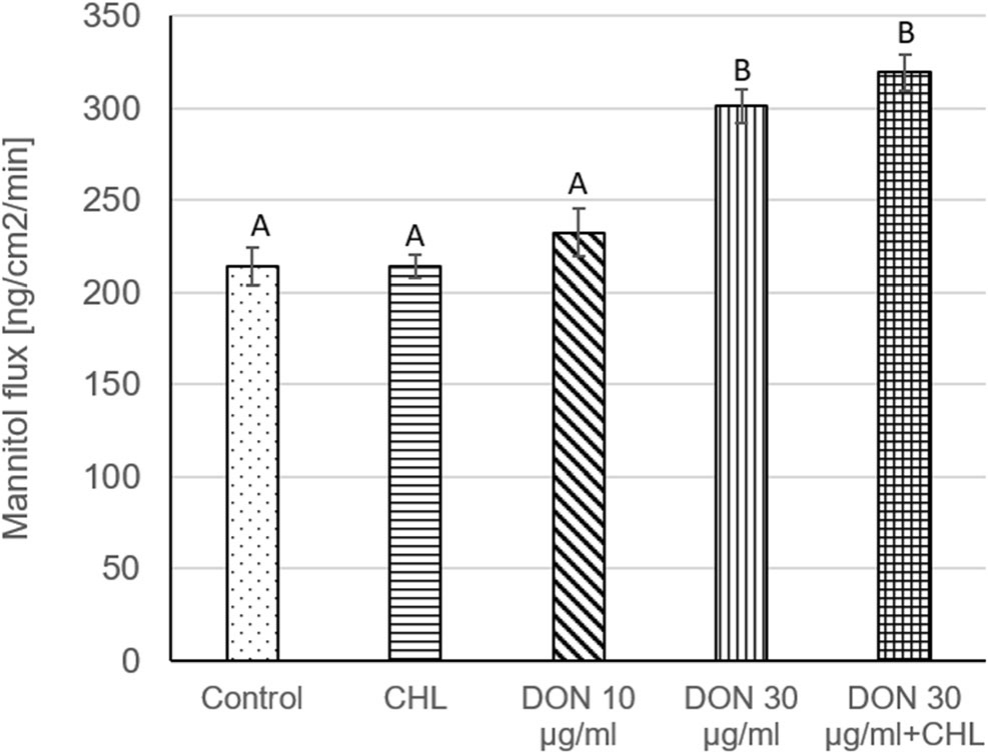
Effect of DON (0, 30, and 90 μg/ml) and DON (90 μg/ml) + CHL (100 μg/ml) on paracellular passage of mannitol across intestinal explants. The rate of mannitol penetration is expressed as the flux (ng/cm^2^/min). Samples for mannitol detection were collected from the serosal reservoir after equilibration (preincubation), 60 and 90 min thereafter. Mannitol flux was calculated for 90 min Data is expressed as mean ± SEM of *n* independent experiments, where *n* = 20 for control, *n* = 6 for CHL, *n* = 8 for DON 30 μg/ml, *n* = 14 for DON 90 μg/ml, and *n* = 6 for DON 90 μg/ml + CHL. The results are considered statistically significant (different letters) when *P* < 0.05

**Fig. 4:**
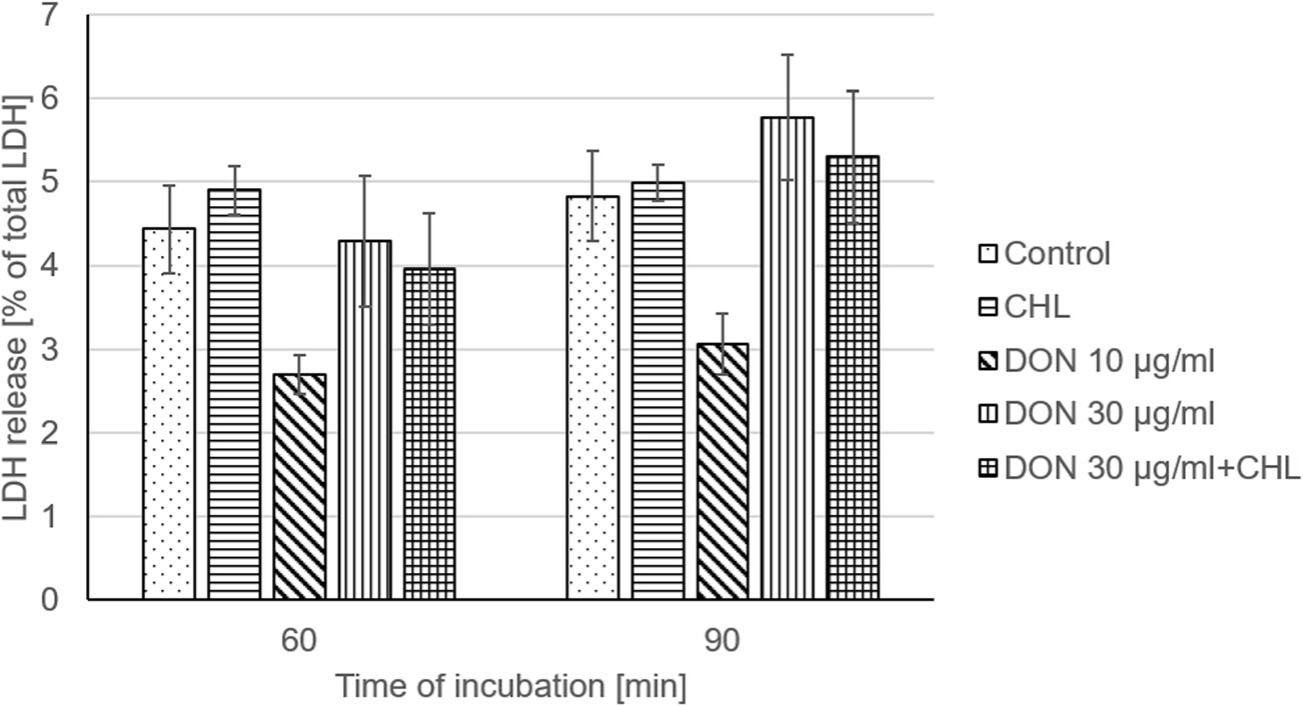
Effect of DON (0, 30, and 90 μg/ml) and DON (90 μg/ml) + CHL (100 μg/ml) on lactate dehydrogenase (LDH) leakage from jejunal mucosa. The results are expressed as % of total LDH which was analyzed after explants homogenization in ice-cold KRB. Samples for LDH measurement were collected from the mucosal reservoir after equilibration (preincubation), 60 and 90 min thereafter. Data is expressed as mean ± SEM of *n* independent experiments, where *n* = 20 for control, *n* = 6 for CHL, *n* = 8 for DON 30 μg/ml, *n* = 14 for DON 90 μg/ml, and *n* = 6 for DON 90 μg/ml + CHL. The results are considered statistically significant (different letters) when *P* < 0.05

To verify the viability and integrity of intestine mucosa preparations, TEER, LY, mannitol, and LDH assays were conducted. Under control conditions (ethanol 0.1%, no addition of DON and CHL), the average TEER reading remained stable during the whole experiment and amounted to 79.7 ± 9.9 Ω cm^2^, i.e., 87.8 ± 5.4% of the initial measurement, at the end of 90-min incubation (Fig. 1). The penetration of LY from luminal into contraluminal compartment was measured to be 343.1 ± 48.4 and 657.2 ± 74.2 ng/ml after 60 and 90 min of incubation without DON addition, respectively (Fig. 2a). The average values corresponded to 0.14 and 0.26% of the initial LY concentration administered to KRB at the mucosal site. The flux of the markers of paracellular transport, i.e., from luminal to contraluminal compartment amounted to 41.6 ± 4.0 and 213.9 ± 10.4 ng/cm^2^/min for LY and mannitol, respectively (Fig. 2b and Fig. 3). The incubation of jejunum explants in KRB did not provoke any remarkable damage of mucosa epithelium cells since the activity of LDH released into the mucosal chamber reached only 4.4 ± 0 and 4.8 ± 0.54% of total LDH after 60 and 90 min of incubation, respectively (Fig. 4). The use of ethanol (0.1%) did not provoke any alteration of measured parameters when compared to data obtained from mucosa explants incubated in KRB without ethanol addition (data not shown).

### The effect of CHL on swine jejunal mucosa explants viability and integrity

The administration of CHL (100 μg/ml) neither induced remarkable impact on the viability nor the integrity of jejunum mucosa explants obtained from adult pigs. The use of CHL did not produce significant decrease of TEER value during 90-min incubation in KRB. The TEER value measured at the end of the experiment amounted to 81.9 ± 2.4% of the initial rate (Fig. 1). Likewise, the flux of the markers of paracellular transport, i.e., LY and mannitol, was not affected by CHL. The magnitude of LY and mannitol flux during 90-min incubation was 42.6 ± 2.3 and 214.1 ± 6.4 ng/cm^2^/min, respectively (Fig. 2b and Fig. 3). Moreover, CHL did not intensify the leakage of LDH from enterocytes since the activity of this enzyme measured in the luminal compartment after 60 and 90 min of explant incubation in KRB amounted to 4.9 ± 03 and 5.0 ± 0.2% of total LDH (Fig. 4).

### The effect of DON on swine jejunal mucosa explants viability and integrity

The use of DON in the concentration of 10 μg/ml did not produce any significant effect on jejunum mucosa explants’ viability or integrity. DON applied in a dose of 10 μg/ml did not cause remarkable drop of TEER value during 90-min incubation. The final TEER reading at the end of the experiment reached 87.5 ± 3.8% of the initial (time 0) measurement (Fig. 1). Similarly, the penetration of paracellular transport markers, LY and mannitol, was not increased in the presence of DON (10 μg/ml). The flux of LY and mannitol from mucosal to serosal site was 43.4 ± 6.2 and 232.3 ± 12.9 ng/cm^2^/min, respectively (Fig. 2b and Fig. 3). Additionally, DON administered in the concentration of 10 μg/ml did not produce any significant leakage of LDH since the activity of the enzyme measured in the mucosal chamber after 60 and 90-min incubation was 2.7 ± 0.2 and 3.1 ± 0.4% of total LDH, respectively (Fig. 4).

On the contrary, the use of DON in the higher concentration, i.e., 30 μg/ml, induced significant increase of mucosa explants’ permeability. The exposure of jejunal preparations to DON (90 μM) for 90 min resulted in a clear drop of TEER value which reached only 37.7 ± 7.8 of the initial value (Fig. 1). Similarly, DON (30 μg/ml) enhanced notably the penetration of LY from luminal into contraluminal compartment during 90-min incubation. The concentration of LY in the serosal chamber was 1591.2 ± 170.1 ng/ml (Fig. 2a). The flux of LY and mannitol in the presence of DON was 90.7 ± 9.7 and 300.9 ± 8.8 ng/cm^2^/min, respectively (Fig. 2b and Fig. 3). Besides, DON administered in the concentration of 30 μg/ml did not intensify LDH leakage into the mucosal compartment. The activity of LDH measured after 60 and 90-min incubation in the presence of the mycotoxin amounted to 4.3 ± 0.8 and 5.8 ± 0.7% of total LDH activity, respectively (Fig. 4).

### The impact of CHL on DON toxicity and transport across swine jejunum explants

Addition of CHL (100 μg/ml) into the incubation chamber filled with KRB containing DON in the concentration of 30 μg/ml did not cause any significant change in TEER value in comparison to trials involving only DON at the same concentration. (Fig. 1). The reading of TEER in the presence of DON+CHL after 90-min incubation was 29.2 ± 7.7% of the initial value, whereas the use of mycotoxin without CHL resulted in TEER measurement of 37.7 ± 7.7% of the initial rate. Alike, there was no remarkable impact of CHL on LY passage through mucosa explants incubated in KRB containing DON (30 μg/ml). The flux of LY calculated for 90-min incubation in the presence of DON or DON+CHL reached 90.7 ± 9.7 and 119.7 ± 9.8 ng/cm^2^/min, respectively (Fig. 2b). Correspondingly, there was no difference in mannitol flux in trials involving DON (30 μg/ml) and DON+CHL usage. The flux of mannitol calculated after 90-min incubation of mucosa explants in either KRB containing DON or DON+CHL amounted to 300.9 ± 8.8 and 319.1 ± 9.8 ng/cm^2^/min, respectively (Fig. 3). Furthermore, the adsorbent did not affect LDH leakage into the mucosal chamber. The activity of LDH in luminal compartment measured after 60- and 90-min incubation was similar in all performed trials, i.e., under control conditions, in the presence of DON (10 and 30 μg/ml) and in the presence of DON+CHL (Fig. 4). The incubation of mucosa explants in KRB containing DON+CHL generated LDH release which reached 4.0 ± 0.7 and 5.3 ± 0.8% of total LDH after 60- and 90-min of incubation, respectively (Fig. 4).

**Fig. 5:**
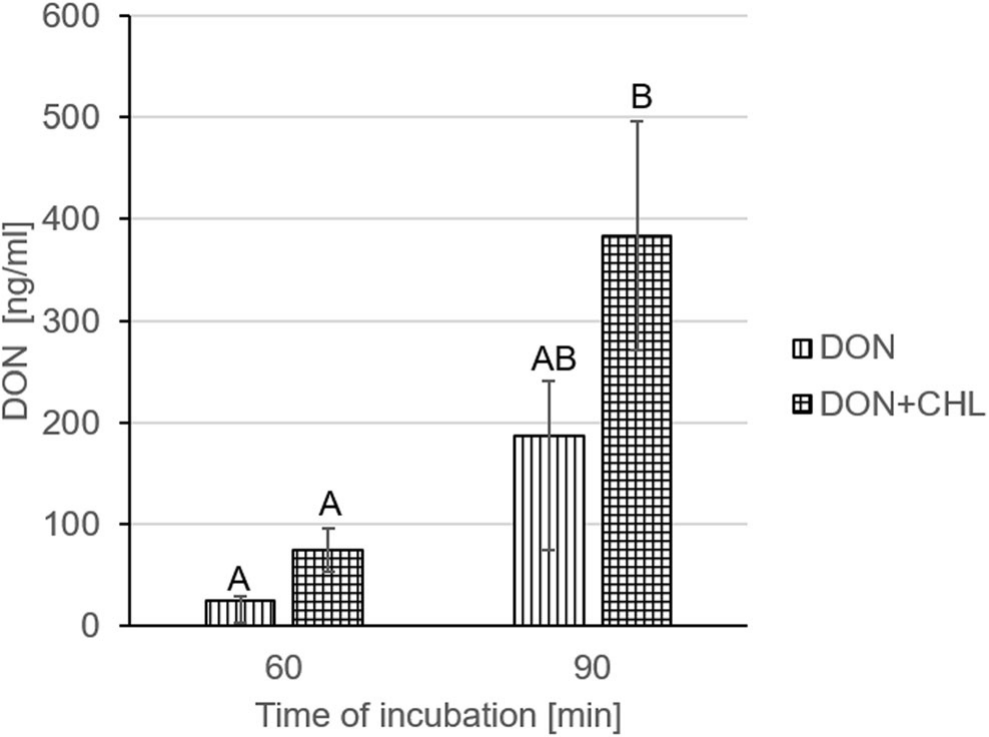
Effect of CHL (100 μg/ml) on DON (90 μg/ml) penetration across swine mucosa explants. The results present the concentration of DON (ng/ml) measured in the serosal compartment after 60- and 90-min incubation in the presence or absence of CHL. Data is expressed as mean ± SEM of 14 (DON 90 μg/ml) and 6 (DON 90 μg/ml + CHL) independent experiments. The results are considered statistically significant (different letters) when *P* < 0.05

Finally, the addition of CHL into the incubation medium did not modify significantly the rate of DON transport across mucosa explants. The concentration of DON measured in the serosal chamber after 60- and 90-min incubation in KRB containing mycotoxin in a dose of 30 μg/ml at the mucosal site reached 24.8 ± 4.4 and 187.1 ± 54.1 ng/ml, respectively (Fig. 5). When CHL was added to the incubation medium supplemented with DON (30 μg/ml), there was no significant change in the penetration of DON into the contraluminal compartment. The concentration of DON measured at the serosal site came to 73.8 ± 21.2 and 384.0 ± 112.4 ng/ml after 60 and 90 min of incubation, respectively (Fig. 5).

## Discussion

Despite many efforts to control the occurrence of fungal toxins in feed and food, mycotoxin contamination is unavoidable and unpredictable. Therefore, new strategies are tested to verify the usefulness of dietary approaches which could reduce the bioavailability of mycotoxins, especially DON. One novel approach includes the use of natural or semi-synthetic compounds of plant origin, e.g., CHL (Boudergue et al. 2009) which was tested in the present study.

In order to obtain reliable results but also to remain in compliance with the 3Rs principles, alternative experimental models based on tissues collected from target animal species should be favored. Hence, the study presented herein was carried out on jejunum mucosa explants isolated from healthy pigs routinely slaughtered at a local abattoir—which makes our study unique. In contrast to numerous trials conducted on jejunum explants isolated from young piglets (Table 1), the intestine samples used to isolate mucosa strips in our studies were obtained from adult pigs (animals of approx. 100 kg of body weight and minimum 6 months old) which might have affected the susceptibility of jejunum preparations to DON. The ex vivo permeability model used to study CHL effect on DON absorption reflects the morphology of the in vivo intestinal epithelium and preserves physiological features essential for absorption studies (Akbari et al. 2014; Westerhout et al. 2014). In particular, the preservation of tight junctions is of major importance due to the proposed mechanism of DON toxicity towards intestine mucosa. The mycotoxin (20–50 μmol/l) is known to activate mitogen-activated protein kinases (MAPKs) pathways and diminish the expression of tight junction protein such as claudin-4 which results in increased paracellular permeability of the intestine and possibly intensified antigen and bacterial translocation from the lumen of the gut (Pestka et al. 2004; Pinton et al. 2009). The significant increase of LY and mannitol translocation across intestinal explants in the presence of DON (30 μg/ml) (Figs. 2 and 3) confirms previous observations of DON (10–50 μmol/l) ability to enhance jejunum permeability to paracellular (Pinton et al. 2009; Akbari et al. 2014; García et al. 2018). Similarly, the decrease of TEER value in the presence of DON noticed herein and previously (Pinton et al. 2009; Halawa et al. 2012; Akbari et al. 2014) proves its negative effect on the integrity of the intestinal explants. On the other hand, the cytotoxic effect of DON was excluded by LDH leakage assay. The use of DON (up to 30 μg/ml) provoked increased LDH leakage neither in swine mucosa explants (Fig. 4) nor in cell models, as described by other authors (Sergent et al. 2006; Pinton et al. 2009; Akbari et al. 2014). Noteworthy, all of the impairments observed in our study refer to DON used in the relatively high dose of 30 μg/ml (90 μmol/l). The trials involving DON application in a lower dose of 10 μg/ml (30 μmol/l), which is comparable to the concentration of DON administered by others (Table 1), did not provoke any significant change of the integrity and permeability of mucosa explants. The use of DON in the concentration of 10 μg/ml reflects realistic exposure of pigs to this mycotoxin The incubation of intestine explants in the presence of 10 μg/ml DON corresponds to animal’s exposure to 9 mg DON/kg feed, assuming that DON diluted in 1 l of gastrointestinal fluid is ingested in one meal and is 100% bioavailable (Pinton et al. 2009). According to the recent scientific opinion of EFSA, the reported levels of DON in feed grains amount up to 9.5 mg/kg (European Food Safety Agency 2017). The difference of the magnitude of the effective doses determined herein (30 μg/ml) and in previous studies (10–50 μmol/l in swine mucosa explants and 5–100 μmol/l in cellular models) (Pinton et al. 2009, 2010; Goossens et al. 2012; Lucioli et al. 2013; Akbari et al. 2014; García et al. 2018) might possibly be explained by a tolerance or compensation of adult pigs to DON (Perlusky et al. 1994; Dänicke et al. 2004) or compensatory mechanisms induced by previous negative effect of DON to re-establish intestinal homeostasis (Pasternak et al. 2018). Based on the results of monitoring studies on feed contamination by mycotoxins in Europe (European Food Safety Agency 2017), it must be assumed that adult pigs had been chronically or continuously exposed to DON before they were slaughtered, whereas piglets used as donors of mucosa explants by others (Table 1) had ingested feed containing controlled doses of DON and only for a relatively short time, as they were slaughtered latest at the age of 3 months.

**Table 1:** Comparison of the age of pigs used as doors of intestine explants, as well as effective concentrations of DON required to observe the toxic effect of the mycotoxin towards jejunum

Age of animals	Concentration of DON necessary to alter mucosa explant permeability	Reference
4 weeks old crossbred piglets	10 μmol/l	Lucioli et al. 2013
4–5 weeks old crossbred piglets	10 μmol/l	Cheat et al. 2015
5 weeks old crossbred castrated male piglets	10 μmol/l	García et al. 2018
2–3 months old of the Deutsches Edelschwein breed male castrated pigs	12–24 μmol/l	Halawa et al. 2012
15–20 kg crossbred piglets	20–50 μmol/l	Pinton et al. 2009
6–7 months old crossbred pigs	90 μmol/l	Present study

The second part of the study included the verification of the effect of a potential adsorbent agent, CHL, on the absorption of DON utilized in the high dose in the Ussing chamber model. CHL previously proved to inhibit AFB_1_ transport across Caco-2 cell monolayer (Mata et al. 2004) and decrease DON concentration (over 85%) in phosphate buffer (Cavret et al. 2010). However, the authors did not verify the ability of CHL to control DON transportation in any absorption model. Unfortunately, the results described herein suggest that CHL neither reduces the intensity of DON transport across mucosa explants nor does it prevent DON-induced impairment of intestine barrier. Data on CHL bioavailability indicate that its uptake by intestinal cells is caused predominantly by a facilitated process, followed by efficient apical efflux of CHL or its derivatives to the luminal compartment, most probably through the activation of ABC transporters (Ferruzzi et al. 2002; Ferruzzi and Blakeslee 2007). Noteworthy, a portion of the internalized CHL and its derivatives is being passed from enterocytes into systemic circulation (Egner et al. 2000). Although the amount of absorbed CHL is never high, it might be sufficient to increase slightly DON penetration through mucosa cells when the complex is formed. Contrary to CHL, DON is not a substrate of the efflux pumps or it has no intracellular access to these membrane transporters. Consequently, its transcellular diffusion into serosal compartment may not be ruled out (Sergent et al. 2006). The results presented herein (Fig. 5) suggest that CHL forms complexes with DON as implied by Cavret et al. (2010), but it does not prevent mycotoxin absorption in the mucosa explant model. On the contrary, to some extent (not remarkable), CHL might intensify DON passage across intestine probably by involving transcellular transport mechanisms. No difference in TEER measurements and LY and mannitol flux between DON and DON+CHL trials supports the hypothesis that CHL does not intensify the paracellular transportation of DON through the explants.

In conclusion, since the breakdown of the tightness of intestine epithelial barrier increases the risk of intestinal and systemic diseases, as well as animal productivity, it should be of major concern to control feed contamination with factors likely to impair intestine integrity, including DON. The results presented herein confirm the negative effect of DON towards pig jejunum mucosa. However, due to the age of pigs used as donors of intestine tissue, the toxic effect of the mycotoxin was observed only when it was used in relatively high doses. Unfortunately, the promising adsorbent agent, CHL, failed to reduce the intensity of DON transport across intestine under in vitro conditions. The received results do not support CHL testing in pigs. Nevertheless, it is of foremost importance to proceed with studies aimed to verify the potency of novel DON detoxifying agents.

## Abbreviations

CHL: Chlorophyllin
DON: Deoxynivalenol
TEER: Transepithelial electrical resistance
LY: Lucifer yellow
LDH: Lactate dehydrogenase
KRB: Krebs-Bicarbonate Buffer
AFB_1_: Aflatoxin B_1_

## References

[CR1] Akbari P, Braber S, Gremmels H, Koelink PJ, Verheijden KA, Garssen J, Fink-Gremmels J (2014). Deoxynivalenol: a trigger for intestinal integrity breakdown. FASEB J.

[CR2] Alassane-Kpembi I, Pinton P, Hupé J-F, Neves M, Lippi Y, Combes S, Castex M, Oswald IP (2018). Saccharomyces cerevisiae Boulardii reduces the deoxynivalenol-induced alteration of the intestinal transcriptome. Toxins.

[CR3] Avantaggiato G, Havenaar R, Viscontia A (2004). Evaluation of the intestinal absorption of deoxynivalenol and nivalenol by an in vitro gastrointestinal model, and the binding efficacy of activated carbon and other adsorbent materials. Food Chem Toxicol.

[CR4] Awad WA, Ghareeb K, Böhm J, Zentek J (2010). Decontamination and detoxification strategies for the Fusarium mycotoxin deoxynivalenol in animal feed and the effectiveness of microbial biodegradation. Food Addit Contam.

[CR5] Bernhoft A, Christensen E, Ivanova LEC, Bergsjø B, Johannessen G (2016) The surveillance programme for feed materials, complete and complementary feed in Norway 2016—Mycotoxins, fungi and bacteria. Norwegian Veterinary Institute, Annual Report 2016

[CR6] Boudergue C, Burel C, Dragacci S, Favrot MC, Fremy JM, Massimi C, Prigent P, Debongnie P, Pussemier L, Boudra H, Morgavi D, Oswald I, Perez A, Avantaggiato G (2009). Review of mycotoxin-detoxifying agents used as feed additives: mode of action, efficacy and feed/food safety. EFSA Supporting Publications.

[CR7] Bryła M, Ksieniewicz-Woźniak E, Waśkiewicz A, Szymczyk K, Jędrzejczak R (2018). Natural occurrence of nivalenol, deoxynivalenol, and deoxynivalenol-3-glucoside in Polish winter wheat. Toxins.

[CR8] Cavret S, Laurent N, Videmann B, Mazallon M, Lecoeure S (2010). Assessment of deoxynivalenol (DON) adsorbents and characterisation of their efficacy using complementary in vitro tests. Food Addit Contam.

[CR9] Cheat S, Gerez JR, Cognié J, Alassane-Kpembi I, Bracarense APFL, Raymond-Letron I, Oswald IP, Kolf-Clauw M (2015). Nivalenol has a greater impact than deoxynivalenol on pig jejunum mucosa in vitro on explants and in vivo on intestinal loops. Toxins.

[CR10] Cheng YH, Weng CF, Chen BJ, Chang MH (2006). Toxicity of different Fusarium mycotoxins on growth performance, immune responses and efficacy of a mycotoxin degrading enzyme in pigs. Anim Res.

[CR11] Dänicke S, Valenta H, Klobasa F, Döll S, Ganter M, Flachowsky G (2004). Effects of graded levels of Fusarium toxin contaminated wheat in diets for fatting pigs on growth performance, nutrient digestibility, deoxynivalenol balance and clinical serum characteristics. Arch Anim Nutr.

[CR12] Dersjant-Li Y, Verstegen MWA, Gerrits WJJ (2003). The impact of low concentrations of aflatoxin, deoxynivalenol or fumonisin in diets on growing pigs and poultry. Nutr Res Rev.

[CR13] Egner PA, Stansbury KH, Snyder EP, Rogers ME, Hintz PA, Kensler TW (2000). Identification and characterization of chlorin e4 ethyl ester in sera of individuals participating in the chlorophyllin chemoprevention trial. Chem Res Toxicol.

[CR14] European Commission 576/2006, Commission Recommendation of 17 August 2006 on the presence of deoxynivalenol, zearalenone, ochratoxin A, T-2 and HT-2 and fumonisins in products intended for animal feeding

[CR15] European Food Safety Agency (2004) Opinion of the scientific panel on contaminants in the food chain on a request from the commission related to deoxynivalenol (DON) as undesirable substance in animal feed. EFSA J 73:1–42. 10.2903/j.efsa.2004.73

[CR16] Knutsen HK, Alexander J, Barregard L, European Food Safety Agency CONTAM Panel (EFSA Panel on Contaminants in the Food Chain) (2017). Scientific opinion on the risks to human and animal health related to the presence of deoxynivalenol and its acetylated and modified forms in food and feed. EFSA J.

[CR17] European Union Register of Feed Additives pursuant to Regulation (EC) No 1831/2003. Edition 3/2018 (262) (Released date 04.05.2018)

[CR18] FDA, (U.S. Food and Drug Administration). Guidance for industry and FDA: Advisory levels for deoxynivalenol (DON) in finished wheat products for human consumption and grains and grain by-products used for animal feed (June 29, 2010; Revised July 7, 2010). Available online: https://www.fda.gov/Food/GuidanceRegulation/GuidanceDocumentsRegulatoryInformation/ChemicalContaminantsMetalsNaturalToxinsPesticides/ucm120184.htm. Accessed on 19.09.2018

[CR19] Ferruzzi MG, Blakeslee J (2007). Digestion, absorption, and cancer preventative activity of dietary chlorophyll derivatives. Nutr Res.

[CR20] Ferruzzi MG, Failla ML, Schwartz SJ (2002). Sodium copper chlorophyllin: in vitro digestive stability and accumulation by Caco-2 human intestinal cells. J Agric Food Chem.

[CR21] García GR, Payros D, Pinton P, Dogi CA, Laffitte J, Neves M, González Pereyra ML, Cavaglieri LR, Oswald IP (2018). Intestinal toxicity of deoxynivalenol is limited by lactobacillus rhamnosus RC007 in pig jejunum explants. Arch Toxicol.

[CR22] Ghareeb K, Awad WA, Böhm J, Zebeli Q (2015). Impacts of the feed contaminant deoxynivalenol on the intestine of monogastric animals: poultry and swine. J Appl Toxicol.

[CR23] Goossens J, Pasmans F, Verbrugghe E, Vandenbroucke V, De Baere S, Meyer E, Haesebrouck F, De Backer P, Croubles S (2012). Porcine intestinal epithelial barrier disruption by the Fusarium mycotoxins deoxynivalenol and T-2 toxin promotes transepithelial passage of doxycycline and paromomycin. BMC Vet Res.

[CR24] Grajewski J, Błajet-Kosicka A, Twarużek M, Kosicki R (2012). Occurrence of mycotoxins in Polish animal feed in years 2006–2009. J Anim Physiol Animal Nutr.

[CR25] Halawa A, Dänicke S, Kersten S, Breves G (2012). Effects of deoxynivalenol and lipopolysaccharide on electrophysiological parameters in growing pigs. Mycotoxin Res.

[CR26] Kabak B, Dobson AD, Var I (2006). Strategies to prevent mycotoxin contamination of food and animal feed: a review. Crit Rev Food Sci Nutr.

[CR27] Karlovsky P (2011). Biological detoxification of the mycotoxin deoxynivalenol and its use in genetically engineered crops and feed additives. Appl Microbiol Biotechnol.

[CR28] Kolf-Clauw M, Castellote J, Joly B, Bourges-Abella N, Raymond-Letron I, Pinton P, Oswald IP (2009). Development of a pig jejunal explant culture for studying the gastrointestinal toxicity of the mycotoxin deoxynivalenol: histopathological analysis. Tox In Vitro.

[CR29] Lucioli J, Pinton P, Callu P, Laffitte J, Grosjean F, Kolf-Clauw M, Oswald IP, Bracarense AP (2013). The food contaminant deoxynivalenol activates the mitogen activated protein kinases in the intestine: interest of ex vivo models as an alternative to in vivo experiments. Toxicon.

[CR30] Mata JE, Yu Z, Gray JE, Williams DE, Rodriguez-Proteua R (2004). Effects of chlorophyllin on transport of dibenzo(a, l) pyrene, 2-amino-1-methyl-6-phenylimidazo-[4,5-b]pyridine, and aflatoxin B1 across Caco-2 cell monolayers. Toxicol.

[CR31] Niderkorn V, Morgavi DP, Pujos E, Tissandier A, Boudra H (2007). Screening of fermentative bacteria for their ability to bind and biotransform deoxynivalenol, zearalenone and fumonisins in an in vitro simulated corn silage model. Food Addit Contam.

[CR32] Pasternak JA, Aka Aiyer VI, Hamonic G, Beaulieu AD, Columbus DA, Wilson HL (2018) Molecular and physiological effects on the small intestine of weaner pigs following feeding with deoxynivalenol-contaminated feed. Toxin 10. 10.3390/toxins10010040 10.3390/toxins10010040 PMC5793127 29329218

[CR33] Perlusky DB, Gerdes RG, Underhill KL, Rotter BA, Jiu PY, Trenholm HL (1994). Effects of low-level dietary deoxynivalenol on haematological and clinical parameters of the pig. Nat Toxins.

[CR34] Pestka JJ, Zhou HR, Moon Y, Chung YJ (2004). Cellular and molecular mechanisms for immune modulation by deoxynivalenol and other trichothecenes: unraveling a paradox. Toxicol Lett.

[CR35] Pinton P, Accensi F, Beauchamp E, Cossalter AM, Callu P, Grosjean F, Oswald IP (2008). Ingestion of deoxynivalenol (DON) contaminated feed alters the pig vaccinal immune responses. Toxicol Lett.

[CR36] Pinton P, Nougayrede JP, del Rio JC, Moreno C, Marin D, Ferrier L, Barcarense AP, Kolf-Clauw M, Oswald IP (2009). The food contaminant, deoxynivalenol, decreases intestinal barrier function and reduces claudin expression. Toxicol Appl Pharmacol.

[CR37] Pinton P, Braicu C, Nougayrede JP, Laffitte J, Taranu I, Oswald IP (2010). Deoxynivalenol impairs porcine intestinal barrier function and decreases the protein expression of claudin-4 through a mitogen-activated protein kinase-dependent mechanism. J Nutr.

[CR38] Rodrigues I, Naehrer K (2012). Prevalence of mycotoxins in feedstuffs and feed surveyed worldwide in 2009 and 2010. Phytopathol Mediterr.

[CR39] Rotter BA (1996). Toxicology of deoxynivalenol (vomitoxin). J Toxicol Environ Health.

[CR40] Sergent T, Parys M, Garsou S, Pussemier L, Schneider YJ, Larondelle Y (2006). Deoxynivalenol transport across human intestinal Caco-2 cells and its effects on cellular metabolism at realistic intestinal concentrations. Toxicol Lett.

[CR41] Sjöberg Å, Lutz M, Tannergren C, Wingolf C, Borde A, Ungell AL (2013). Comprehensive study on regional human intestinal permeability and prediction of fraction absorbed of drugs using the Ussing chamber technique. Eur J Pharm Sci.

[CR42] Smith MC, Madec S, Coton E, Hymery N (2016). Natural co-occurrence of mycotoxins in foods and feeds and their in vitro combined toxicological effects. Toxins.

[CR43] Streit E, Schatzmayr G, Tassis P, Tzika E, Marin D, Taranu I, Tabuc C, Nicolau A, Aprodu I, Puel O, Oswald IP (2012). Current situation of mycotoxin contamination and co-occurrence in animal feed—focus on Europe. Toxins.

[CR44] Streit E, Naehrer K, Rodrigues I, Schatzmayr G (2013). Mycotoxin occurrence in feed and feed raw materials worldwide: long-term analysis with special focus on Europe and Asia. J Sci Food Agric.

[CR45] Ungell AL, Andreasson A, Lundin K, Utter L (1992). Effects of enzymatic inhibition and increased paracellular shunting on transport of vasopressin analogues in the rat. J Pharm Sci.

[CR46] Westerhout J, van de Steeg E, Grossouw D, Zeijedner EE, Krul CAM, Verwei M, Wortelboer HM (2014). A new approach to predict human intestinal absorption using porcine intestinal tissue and biorelevant matrices. Eur J Parm Sci.

[CR47] Young JC, Zhou T, Yu H, Zhu H, Gong J (2007). Degradation of trichothecene mycotoxins by chicken intestinal microbes. Food Chem Toxicol.

